# Mechanical Innovations of a Climbing Cactus: Functional Insights for a New Generation of Growing Robots

**DOI:** 10.3389/frobt.2020.00064

**Published:** 2020-06-09

**Authors:** Patricia Soffiatti, Nick P. Rowe

**Affiliations:** ^1^Department of Botany, Federal University of Parana State (UFPR), Curitiba, Brazil; ^2^AMAP, Univ Montpellier, CIRAD, CNRS, INRAE, IRD, Montpellier, France

**Keywords:** biomechanics, climbing cactus, indeterminate growth, light architecture, searcher, soft robotics, two-step attachment

## Abstract

Climbing plants are being increasingly viewed as models for bioinspired growing robots capable of spanning voids and attaching to diverse substrates. We explore the functional traits of the climbing cactus *Selenicereus setaceus* (Cactaceae) from the Atlantic forest of Brazil and discuss the potential of these traits for robotics applications. The plant is capable of growing through highly unstructured habitats and attaching to variable substrates including soil, leaf litter, tree surfaces, rocks, and fine branches of tree canopies in wind-blown conditions. Stems develop highly variable cross-sectional geometries at different stages of growth. They include cylindrical basal stems, triangular climbing stems and apical star-shaped stems searching for supports. Searcher stems develop relatively rigid properties for a given cross-sectional area and are capable of spanning voids of up to 1 m. Optimization of rigidity in searcher stems provide some potential design ideas for additive engineering technologies where climbing robotic artifacts must limit materials and mass for curbing bending moments and buckling while climbing and searching. A two-step attachment mechanism involves deployment of recurved, multi-angled spines that grapple on to wide ranging surfaces holding the stem in place for more solid attachment via root growth from the stem. The cactus is an instructive example of how light mass searchers with a winged profile and two step attachment strategies can facilitate traversing voids and making reliable attachment to a wide range of supports and surfaces.

## Introduction

Plants have recently become a focus of interest as potential bioinspired models for soft robotics (Mazzolai et al., 2014; Walker, 2015; Mazzolai, 2017; Del Dottore et al., 2018). Plants offer a rich source of potential innovations because of (i) their many kinds of growth and movement by growth (ii) their modular construction and strategically positioned functional structures and specialized organs and (iii) their morphological and functional plasticity—in particular their ability to make simple changes in developmental to profoundly change structural properties and functionality. The combination of these life history processes bestows high levels of adaptability in biological contexts and thus offers a wide adaptive potential for technological applications. Climbing plants possess a number of traits that can deal with many environmental challenges that other kinds of robot cannot (Walker, 2015). The approach seeking bioinspiration from the whole organism and its life history and adaptive strategies has recently been coined as an “organism-inspired” approach as opposed to inspiration on finer cellular and molecular levels (Del Dottore et al., 2018).

Research on different plant growth forms and life histories has highlighted the architectural e.g., (Barthélémy and Caraglio, 2007), developmental e.g., (Speck and Rowe, 1999; Bateman, 2002), and mechanical e.g., (Rowe and Speck, 2005) diversity of different plant growth forms and the mechanisms that underline them. Climbing plants represent some of the most developmentally complex and potentially plastic growth forms (Rowe, 2018). They have highly indeterminate growth patterns, exceptionally variable stem mechanics and highly adaptive behavior as erect “searchers” or creeping, climbing or pendulous stems (Putz and Mooney, 1991). The climbing habits have evolved many times in plants (Gentry, 1991) and their stem morphology, biomechanics, climbing modes, attachment organs, roots, and anatomy are immensely diverse. Climbing plants are now providing a lot of choice for bioinspired technologies in robotics (Mehling et al., 2006; Wooten and Walker, 2016, 2018; Fiorello et al., 2018, 2019, 2020; Wooten et al., 2018; Must et al., 2019).

Over recent decades, plant biomechanics research has explored much of the diversity of form and function in both living and fossil plants and has documented the structural novelties that have appeared during evolution e.g., (Speck and Rowe, 2001). Today, plant biomechanics is an intrinsic part of the research for bioinspired technological solutions. Many of these investigations have yielded fascinating insight into the functioning of individual plant structures and organs at all scales and hierarchical levels, many of which could be of potential interest for bioinspired applications in robotics. This has proved an important source of bioinspiration in many applications: light weight fabricated plant stem-like organizations (Milvich et al., 2006); self-repairing and self-healing structures and technologies (Huss et al., 2018a,b; Speck et al., 2018; Yang et al., 2018); sealing foam for pneumatic systems based on the strategy found in the stems of *Aristolochia* (Busch et al., 2010; Rampf et al., 2011, 2013). The variety of attachment structures and organs of climbing plants is remarkable and has a great potential for bioinspired anchoring devices (Melzer et al., 2010; Bauer et al., 2011; Andrews and Badyal, 2014; Gallenmuller et al., 2015; Burris et al., 2018; Fiorello et al., 2018, 2019; Must et al., 2019). Plant surfaces have also offered inspiration for several technological applications for the creation of self-cleaning and water-repellent materials (Neinhuis and Barthlott, 1997; Koch and Barthlott, 2009; Latthe et al., 2014; Barthlott et al., 2017).

Bioinspired studies based on plants are now increasingly turning toward soft robotic applications (Mazzolai et al., 2014; Mazzolai, 2017). A turning point is that movement via adaptive growth is being integrated into bioinspired designs for artifacts to grow and move by artificial growth and movements like plant stems (Hawkes et al., 2017), tendrils (Must et al., 2019), tendril and searcher like structures (Mehling et al., 2006; Wooten and Walker, 2018; Wooten et al., 2018) and roots (Sadeghi et al., 2014, 2016; Del Dottore et al., 2019). The approach also considers how plants develop and adapt their modes of growth and varying stem stiffness and rigidity to locate supports as well as attaching and climbing on different substrates.

Plant biodiversity provides a diverse tableau from which to choose potential kinds of bioinspired robotic behavior. Furthermore, convergent and parallel evolution has provided a wide choice of different biological models—different “ways” of being a root or stem or tree or climbing plant. Steven Vogel summarized nicely the fact that convergence can be a source of confusion for evolutionary biologists but a bonus for bioinspired research: “for the biologist and evolutionist convergence is a fascinating… problem, for the bioinspired researcher, convergence and diversity is a… gift” (Vogel, 2003).

Cacti are well-known for their remarkable adaptations to arid and semi-arid conditions (Gibson and Nobel, 1986), resulting in a reduced structural organization, where leaves are absent and succulent stems take over photosynthesis and provide storage, in parallel with support and water conduction. The focus of this study is a root-climbing cactus, a rather unusual growth form that shows truly indeterminate growth with behavioral and morphological shifts that change according to the kind of 3-dimensional environment. The combination of succulence, creeping and climbing is rare in such plants. Previous studies have shown how upright cactus stems have unusual anatomies but can still attain relatively large sizes and heights (Niklas et al., 1999). Furthermore, certain very rare forms have an adapted organization for creeping along the ground (Niklas et al., 2003).

Many climbing plant stems are merely millimeters in diameter during searching and initial climbing. Scaling up geometries and properties will be necessary if biological principles from diminutive plants are to be applied to robotic artifacts constrained by size limits of available technologies. This is especially crucial for reaching across voids and avoiding buckling. Scaled-up structures must be still capable of adaptively keeping on course toward the desired support e.g., (Del Dottore et al., 2019) and minimizing constructional cost and mass in terms of energy and materials. We investigated developmental changes of a cactus species that develops a large diameter stem via primary growth of soft tissues. These reach a similar size (centimeters in diameter) as some current robotic artifacts e.g., (Sadeghi et al., 2014, 2016).

In this paper we outline an approach for identifying functional traits of a climbing plant in its natural habitat and how these are linked to the biomechanics and basic organization of the stem. To our knowledge this is the first account of the life history of a climbing cactus that includes biomechanical observations. Our goal was to identify its behavioral characteristics that allow it to navigate through highly unstructured, heterogeneous and even moving environment. In this first paper we highlight the key developmental features, how the plant deploys them and provide a summary of features that can be examined and potentially integrated into technological projects.

## Materials and Methods

*Selenicereus setaceus* (Cactaceae) is a climbing cactus with succulent, leafless stems occurring in dry coastal formations of Atlantic Forest in Southern Brazil. Plant stems were collected from the “Restinga” coastal lowland dry forests in Armação dos Buzios town (22° 44′ 49″ S, 41° 52′ 54″ W, 174 km from Rio de Janeiro city), Rio de Janeiro State. The region is composed of a mosaic of vegetation types and the climate is typically warm and dry. The so-called “Restinga forests” are lowland dry forests composed of trees and shrubs, where *S. setaceus* is a common component.

We outline a methodological approach, which can serve as a working model for detecting biological traits for potential technical solutions in robotics. The methodology can be viewed as several steps: (a) Observing the ecological context and variability of functional traits in the natural habitat such as searching, climbing and creeping behavior. All of these represent potentially interesting features for enabling robotic artifacts to traverse different kinds of terrain and crossing voids. (b) We then outline how the mechanical properties of these different behaviors are modified by different combinations of tissues during growth and development. This step is of interest for adapting and fine-tuning properties via simple changes in geometry and material composition. (c) We then compare the relative “cost” of different mechanical solutions by comparing rigidity and stiffness with the carbon content or biomass that has been attributed by the plant for different roles. (D) Finally, we list the biological functions and discuss their relevance for bioinspired robotics research using, as far as possible, a biologically non-technical language. This overall approach represents just one example of how the study of a biological system might be focused on the search for functional attributes useful for novel technologies for robotics applications.

### Ecological Observations

We made observations at ~12 sites where *S. setaceus* was common and presented its full range of growth phases from creeping and climbing to searching stems. Our main aim was to gather observations about its growth through complex three-dimensional spaces from forest floor, to low level branches and scrub to climbing stems on tree trunks and as searcher stems emerging from the forest margin and tree canopies. We measured the reach of 34 vertical to horizontal searcher stems as the length of stem segment from the last supported point on the host vegetation to the apex of the searcher. We did not include stems that had reached or passed their maximum length as self-supporting axes.

### Mechanical Tests and Macro Anatomy

Three different types of stem were collected from natural sites for the mechanical tests and kept in humid conditions prior to the measurements. Three-point bending tests (Vincent, 1990) were carried out on the stems using a portable Instron machine (In-Spec 2200, Instron Corporation, Norwood, MA, USA; http://www.instron.com). The study included a total of 127 stem segments: 36 basal stem segments that were approximately cylindrical in cross-section (from 25 individuals); 62 stem segments that were approximately triangular in cross-section (from 32 individuals) and 34 apical “searcher” stems that were approximately star-shaped in cross-section (from 28 individuals). We obtained a span-to-depth ratio of 20–30 after tests were taken to determine the minimum span to depth ratio necessary to minimize the influence of shear on the measured bending (Vincent, 1990).

Flexural rigidity (*EI*) is the resistance of the stem to a bending force. This parameter is the product of the axial second moment of area (*I*) (cross section dimensions and shape), and the elastic modulus (*E*) of the stem.

(1) $$\begin{array}{l} \text{Flexural rigidity}=E^{*}I \end{array}$$

Stem flexural rigidity (Nmm^2^) was calculated as:

(2) $$\begin{array}{l} EI=L^{3*}b/48 \end{array}$$

where L is the distance (mm) between the supports in three-point bending and *b* is the slope of the force-deflection curve (Nmm^−1^).

The second moment of area (*I*) is a geometrical property of a cross section that describes the spatial distribution of areas within the section with reference to the centroid and neutral axis in bending.

The cross-sections of stems showed highly complex cross-section geometries that varied from circular to triangular to highly winged or star-shaped. Following each mechanical test, we prepared three transverse slices from the middle 5 cm portion of each tested stem segment, each slice was then orientated in the direction of the applied force during the test and then photographed. Image analysis software “Optimas”, V.6.5.172, Media Cybernetics, Inc., Rockville, MD, USA was then used to trace the transverse outlines of each slice and a macro (courtesy of T. Almeras, Montpellier) was then used to calculate the second moment of area of each stem shape.

Young's modulus is the stiffness of a material, defined as the slope of the linear region in a stress-strain curve. It is a parameter used for homogeneous solid materials. Taking into consideration that plant stems are significantly anisotropic structures composed of different tissues with distinct properties, the concept of a Young's modulus refers here to a composite structure in bending. In the literature it has been previously referred as a “structural” Young's modulus (Speck and Rowe, 1999).

Young's modulus in bending *E* (MNm^−2^) was obtained using the following formula:

(3) $$\begin{array}{l} E=EI/I \end{array}$$

where *EI* (Nmm^2^) is the flexural rigidity of the stem (Equation 1) and *I* (mm^4^) is the axial second moment of area of the stem calculated using Image analysis software.

### Dry Matter Content and Dry Matter Concentration

A fresh portion of each tested stem was used to calculate the dry matter content [DMC, dry mass (mg)/fresh mass (g)] and the dry matter concentration (D, dry mass/volume), also called density (Pérez-Harguindeguy et al., 2013). For the volume calculation we used the weight-displacement method (Hughes, 2005). We first obtained the fresh weight using a digital balance. The sample was then attached to a fine needle and immersed in a water-filled container placed on a digital balance without touching the walls of the container. The weight of the water displaced was measured, which corresponds to the volume of the sample in cm^3^. For the dry weight, we left the sample to dry in a laboratory oven under forced-air ventilation for ca. 15 days, until reaching a constant weight. With the obtained values we calculated the DMC and the D for comparing the different stems biomass. We used a total of 90 stem segments: 32 circular, 35 triangular and 23 star-shaped.

### Statistical Analysis

All quantitative data were analyzed using non-parametric Kruskal-Wallis tests. followed by Dunn's *post hoc* tests (Siegel and Castellan, 1988) in software Statistica (StatSoft, Inc., 2013). Non-parametric tests were chosen for comparing cross-sectional areas, second moments of area, flexural rigidity and Young's modulus between the stem types—circular, triangular, and star-shaped. We elected to use the non-parametric K-W test followed by the relatively conservative *post-hoc* Dunn's test because: (a) some of the tested parameters were not normally distributed for each stem category, (b) degrees of variance for some parameters were not very homogeneous (as is often the case for geometrical and mechanical values), and (c) because of difficulties of sampling equivalent numbers for each stem type. Finally, from a biologically point of view, non-parametric rank-based comparisons are arguably more suitable for comparisons of stem properties that are developmentally ranked rather than as elements belonging to separate entities.

## Results

### Ecology and Phases of Growth

**Figure 1:**
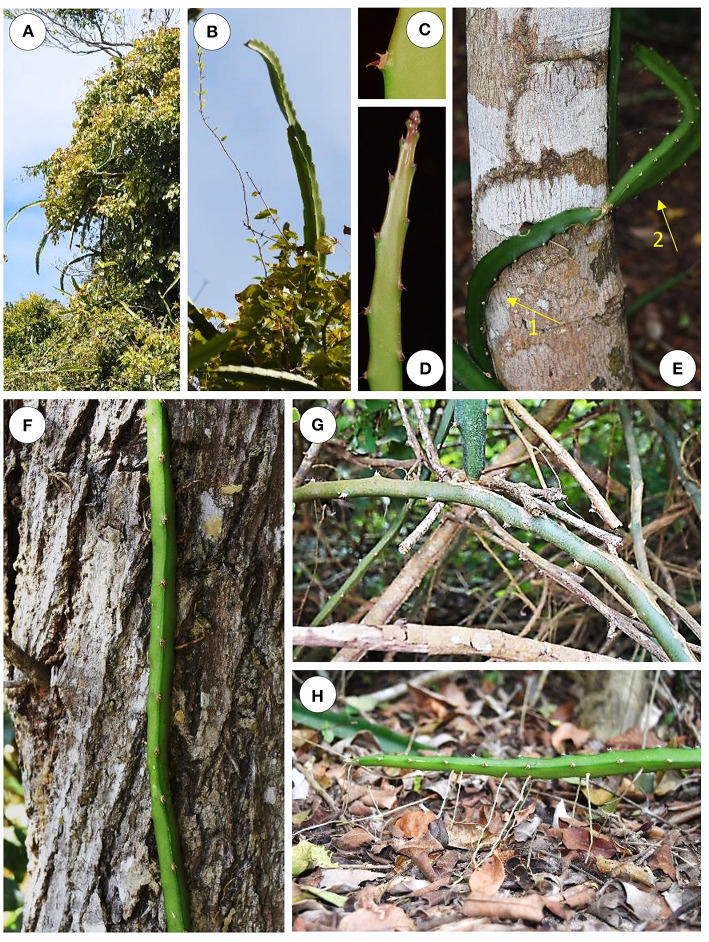
Growth habits of *Selenicereus setaceus* (Cactaceae). **(A)** The species is a tree climber and young apical stems are adapted as “searchers” and emerge from the tree branches. **(B)** Searchers have a star-shaped or winged profile and can grow vertically or horizontally in search of supports. **(C)** Detail of multi-angled clusters of recurved spines; these occur along the edges of three-ridged stems. **(D)** Young searcher apex: spines are initially pointing toward the tip and flattened against the stem; they become recurved during later development and are deployed so as to attach in several directions. **(E)** Triangular root-climbing stems partly coiling around a tree trunk; the more basal portion of the stem is attached by spines and roots to the trunk (arrow 1) while the apex is searching freely (arrow 2). **(F)** A triangular stem climbing up a tree trunk initially deployed recurved hooks that grappled onto the support prior to the development of stronger anchorage by roots. **(G)** Older, basal cylindrical stem traversing complex understory of surrounding stems and branches. **(H)** Roots also attach to the soil at ground level.

*Selenicereus setaceus* exists commonly as a climbing plant in the “Restinga forest” of southern coastal Brazil. It shows highly variable behavior according to the age and position of the plant and in relation to its immediate environment (Figures 1A–H). It was observed on a wide variety of substrates: creeping along the forest floor (Figure 1H), climbing among canopy branches of scrub and trees (Figure 1A) and climbing up tree trunks to 8 m high (Figure 1F). Older, basal stems are circular to elliptical in cross-section with a smooth, brown to green surface (Figure 1G). They gave rise to the green, photosynthetic climbing stems. These stems are triangular to rounded triangular in cross section—often producing roots which adhere to a wide range of surfaces. Younger stem branches we termed “searchers” (Figure 1B) are light green photosynthetic stems bearing spines. These have a “winged,” triangular to star-shaped cross-section and often emerge from trees to cross gaps toward neighboring supports. All categories of stem bear groups of recurved spines. In the young tips of growing stems, spines are initially pointing apically and flattened against the stem (Figure 1D); during development they become generally recurved and point in several directions (Figure 1C).

### Two-Step Attachment Strategy

**Figure 2:**
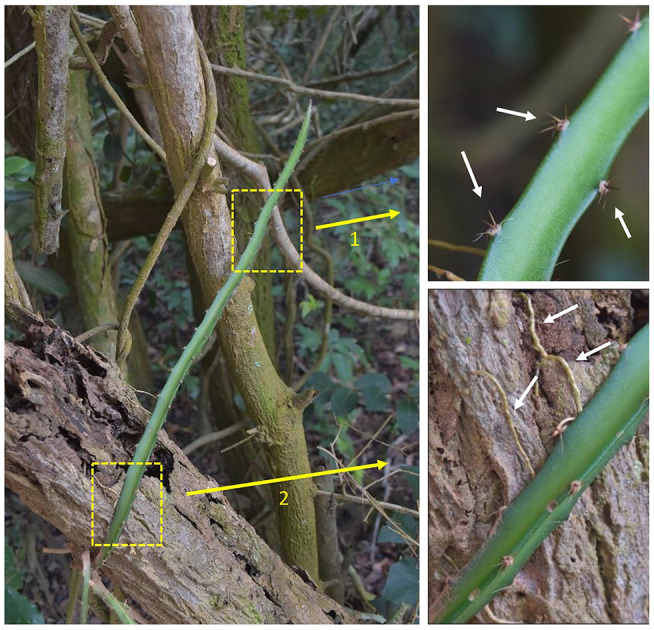
Two-step attachment strategy. The climbing cactus employs a two-step strategy for attaching to a variety of substrates. (1) Initial grappling-steadying attachment using clusters of recurved spines (arrow 1) fixing the plant stem in place while it grows and (2) Solid anchoring using lateral roots (arrow 2) in addition to resource gathering (e.g., water). The strategy is of interest for growing robots as it will allow a device to hook on to the surrounding unstructured environment providing temporary support (bracing) for growth and securing a more solid fixation even in potentially moving (e.g., wind-buffeted) environments. White arrows indicate clusters of multi-angled, recurved spines, which are themselves arranged at multi-angles on the plant stem (top right), and roots invading the crevices on the tree surface (bottom right).

This species employs a “two-step” attachment strategy. In a first step, the clusters of lateral spines (2–5 mm long) act as grappling hooks which fix the searching and climbing stem in place (Figure 2 arrow 1). The groups of 3 to five spines per group and their arrangement on the three angled stem ridges are highly effective for initial adhesion in a wide range of three dimensionally structured environments from laterally clinging to tree trunks and branches (Figure 2 arrow 1), small diameter twigs in the moving tree canopy as well as friable soil surfaces and even stone and concrete objects. Our field observations in a range of environments suggest that this spine attachment serves to mechanically stabilize and fix long searcher stems that would otherwise risk sliding away from supports during early stages of climbing growth. The Restinga forest is a coastal, predominantly wind-exposed environment and we observed that spine attachment was likely important for retaining attachment and stability for stems climbing into the finer branches of trees and scrub.

In a second step (Figure 2 arrow 2) stems that have already been stabilized by hooks and that are held in place become more firmly attached to the support via roots that emerge from the stem surface and grow over the surface of the support entering any fissures or spaces. Stems that are held firmly in place in this way can then continue growth apically without the risk of bending or twisting away from the support and falling to the ground.

### Stem Biomechanics and Geometry

**Figure 3:**
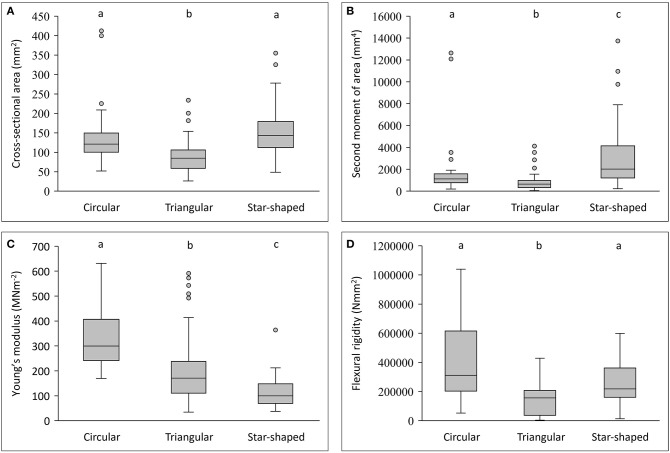
Box plots of geometry and mechanical properties of stem types in *Selenicereus setaceus* (circular, triangular and star-shaped). **(A)** Cross-sectional areas (mm^2^): circular and star-shaped stems have similar cross-sectional areas that are generally larger than climbing triangular stems. **(B)** Second moment of areas (mm^4^): star-shaped stems show the largest second moment of areas for an equivalent surface area due to their highly winged shape. **(C)** Young's modulus (MNm^−2^): stems decrease in stiffness from old (circular) to young stems (star-shaped). **(D)** Comparison of flexural rigidity (Nmm^−2^): star-shaped stems reach a high rigidity, approaching the values for circular stems but retain a “light” structural organization. Inner lines: medians; boxes: 25th and 75th percentiles; whiskers: max and min values; excluding outliers: circles (not depicted in **D**); small letters show significant differences (Dunn's *post hoc* test) between median values of stem types.

**Figure 4:**
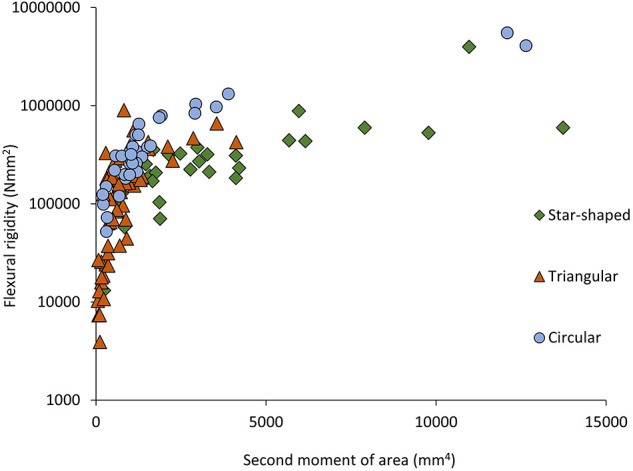
Flexural rigidity (Nmm^2^) plotted against second moment of area (mm^4^) for the three stem types of *Selenicereus setaceus* (circular, triangular and star-shaped): star-shaped stems reach high values for rigidity due to their higher values of second moment of area (n_circ_ = 36; n_tri_ = 62; n_star_ = 34).

**Figure 5:**
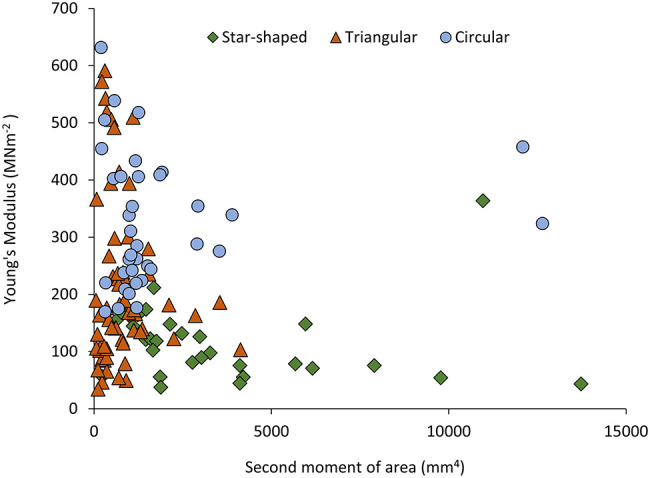
Young's Modulus (MNm^−2^) plotted against second moment of area (mm^4^) for the three stem types of *Selenicereus setaceus* (circular, triangular and star-shaped): highest values of stiffness are found in the circular stems, followed by intermediate values in the triangular and the lowest values in star-shaped searcher stems (n_circ_ = 36; n_tri_ = 62; n_star_ = 34).

**Figure 6:**
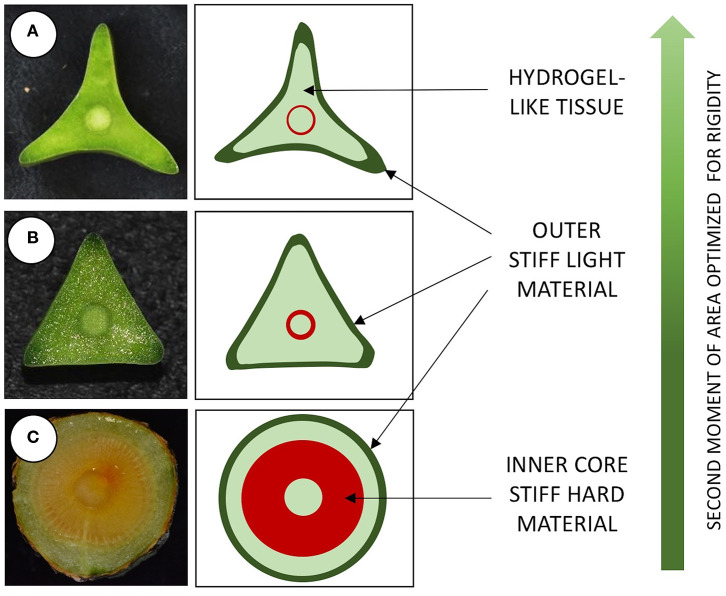
Structural organization of three stem types in *Selenicereus setaceus* from different phases of growth. **(A)** Apical searcher stems are star-shaped in cross-section and have a light organization with: an external layer of stiff light tissue, a soft inner bulking tissue exuding viscous hydrogel-like matrix surrounding; a thin central cylinder of stiffer wood tissue; a central core of soft tissue (pith). **(B)** Root-climbing stems are triangular in cross-section and composed of a similar outer stiff material, inner soft cortex, wood cylinder and pith. **(C)** Basal circular stems are circular in cross-section, with an outer layer of stiff light material, a narrower layer of soft cortex and a broader cylinder of stiff tissue (wood); Circular stems are stiffer in terms of Young's modulus compared to younger triangular and star-shaped stems because of the larger proportion of wood within the stem. The star-shape morphology of searcher stems greatly improves the second moment of area, optimizing rigidity and thus guaranteeing support to traverse voids of up to 1 m.

Stems differed in mechanical properties, cross-sectional geometry and tissue composition (Figures 3A–D, 4–6). Generally, flexural rigidity (*EI*) was higher (for a given stem size) in basal, circular stems (641,683 ± 1,081,766 Nmm^2^) than both triangular climbing stems (177,804 ± 172,821 Nmm^2^) and apical searcher stems (376,712 ± 664,598 Nmm^2^) (*P* = <0.05) (Figure 4).

This pattern was also observed in terms of Young's modulus with rounded stems showing higher values of Young's modulus (328 ± 115 MNm^−2^) than younger climbing (214 ± 143 MNm^−2^) and searcher stems (114 ± 64 MNm^−2^) (Figure 5). Second moment of area varied significantly from older basal rounded stems (1875 ± 2720 mm^4^) and was significantly smaller in triangular stems (799 ± 780 mm^4^) but tended to be larger in the star-shaped searcher axes (3322 ± 3190 mm^4^) (Figures 3B, 4–5). Star-shaped stems reached high values of rigidity (*EI*) approaching that of circular stems due their high values of second moment of area resulting from their winged geometry (Figure 4). We calculated by how much the circular, triangular and star-shaped cross-sections increased in second moment of area compared with theoretical, exactly circular cross-sectional areas for each stem (Table 1). Star-shaped stems showed a significant increase in second moment of area of 67% compared with an equivalent circular model, while triangular and circular stems showed much lower increases of 30 and 27%, respectively (Table 1). In general, the stems present relatively low values of stiffness (*E*), where triangular stems present intermediate values between circular (highest) and star shape stems (lowest) (Figures 3C, 5).

**Table 1:** Cross sectional areas and second moments of area (*I*) of a circle of equivalent area and second moment of area (*I*) measured using the Image software Optimas of three stems types of *Selenicereus setaceus*: circular, triangular and star-shaped (means) (n_circ_ = 36; n_tri_ = 62; n_star_ = 34).

**Stem type cross-section**	**Total cross sectional area (mm^**2**^)**	***I* for a circle of equivalent area (mm^**4**^)**	***I* measured (mm^**4**^)**	**Percentage gain in *I* (%)**
Circular	136.22	1477.46	1875.31	26.93
Triangular	87.70	614.56	798.33	29.90
Star-shaped	157.08	1967.86	3322.25	68.83

### Tissue Composition and Dry Matter Content

Tissue contributions to cross-sectional area (% *A*) and second moment of area (% *I*) varied significantly between different stem types (Table 2). All stem types had a large proportion of cortex (including outer collenchyma, epidermis and/or periderm), especially star-shaped stems, which showed 88% of the total cross-sectional area occupied by these tissues. Dissection of stems for mechanical tests indicated that the soft cortex exuded high levels of hydrogel-like mucilage that expanded in contact with water. Older circular stems showed the highest percentage cross-sectional areas of wood cylinder reaching 15%, while the wood cylinder in triangular and star-shaped stems reached only 5 and 4%, respectively of the cross-sectional area. The percentage of pith area did not vary significantly between stem shapes.

**Table 2:** Percentage contribution of tissues to cross-sectional area (CSA) and second moment of area (*I*) of three stems types of *Selenicereus setaceus*: circular, triangular and star-shaped (means ± standard deviations, tested with Kruskal-Wallis (*P* < 0.001) and Dunn's *post hoc* tests (*P* < 0.05).

**Stem type cross-section**	**Cortex and epidermis/peridermis**	**Wood cylinder**	**Pith**
**Contribution to CSA (%)**			
Circular	77.49 (±6.33)^A^	14.71 (±6.40)^A^	7.79 (±2.39)^A^
Triangular	86.29 (±4.28)^B^	4.75 (±1.42)^B^	8.96 (±3.61)^A^
Star-shaped	88.03 (±2.16)^C^	4.38 (±0.67)^C^	7.59 (±1.64)^A^
**Contribution to** ***I*** **(%)**			
Circular	94.07 (±2.93)^A^	5.17 (±2.96)^A^	0.77 (±0.34)^A^
Triangular	98.05 (±1.03)^B^	1.08 (±0.54)^B^	0.86 (±0.57)^A^
Star-shaped	98.56 (±0.71)^C^	0.81 (±0.35)^C^	0.63 (±0.37)^A^

*Different letters indicate significant differences between groups (n = 10)*.

Tissue contributions to second moment of area varied significantly between stem types (Table 2). The contribution of cortex (including outer collenchyma, epidermis and/or periderm) dominated the second moments of area in all stems, reaching 94% in circular stems, 98% in triangular and 99% in star-shaped stems. In contrast, wood cylinder contribution was very low, only 5% in circular stems, 1% in triangular stems and <1% in the star shape stems. The pith showed <1% of contribution in all stem types.

All values for dry matter content, density and percentage of biomass were significantly different between the stem types (Table 3). Biomass content was highest in circular basal stems because of the higher amount of denser, lignified wood and presence of a periderm.

**Table 3:** Dry matter content. density and percentage of biomass of three stem types of *Selenicereus setaceus*: circular. triangular and star-shaped (means ± standard deviations).

**Stem type cross-section**	**Dry matter content (mg.g^**−1**^)**	**Density (g.cm^**−3**^)**	**Percentage of biomass**
Circular	154.13 (± 41.81)^A^	0.15 (± 0.04)^A^	15.41 (±4.18)^A^
Triangular	108.33 (± 24.06)^B^	0.1 (± 0.02)^B^	10.83 (±2.41)^B^
Star-shaped	90.41 (±41.21)^C^	0.09 (± 0.04)^C^	9.0 (± 4.12)^C^

*Tested with Kruskal-Wallis (P < 0.001) and Dunn's post hoc tests (P < 0.05). Different letters indicate significant differences between groups (n_circ_ = 32; n_tri_ = 35; n_star_ = 23)*.

## Discussion

### The Climbing Cactus as a New Concept Generator for Growing and Climbing Robot Technologies

The stem biomechanics in climbing and searching stems of this plant are characterized by a light-biomass architecture of thin-walled tissue which needs to be turgescent to retain stiffness. Basal (circular) parts of the plant are constructed of stiffer tissues than more apical parts. This organization differs mechanically from many vines and lianas in which basal segments are highly flexible (Speck and Rowe, 1999; Speck et al., 2003; Rowe et al., 2004, 2006; Rowe and Speck, 2005). The combination of succulence and turgescence, searchers with star-shaped geometries as well as a two-step attachment strategy, provides a novel combination of functional traits that might be considered as potential innovations for new climbing plant-like robots. We discuss these as concept generators in terms of overall organization, reaching across voids and attachment (Box 1).

Box 1 **Overview of biological structures and their biological functions in heterogeneous environments, mechanical organization across the plant body and conceptual applications for growing robotic artifacts.**

**Table:** 

**Biological structure**	**Biological function in habitat**	**Mechanical organization**	**Concept for applications**
Stem geometry circular, triangular to star-shaped	For adapting stem profiles for modulating mechanical properties and creeping, climbing and searching, while minimizing physiological and material “costs.”	Gradient of profile geometries from circular (base) to deeply lobed at apex optimized for rigidity in searcher stems.	Growth process adapts profiles for different phases of growth and navigation. e.g., load-bearing searchers with optimized star-shapes using light material architectures.
Reflexed multi-directional spine clusters	Grappling on highly variable shapes and sizes of support and substrate. Step 1 attachment for initial steadying and bracing for facilitating solid attachment (step 2).	Smart (preformed) deployment from folded position at stem tip to open and multi-directional clusters lower down stem.	Incorporation of multi-directional grappling clusters arranged in different directions for attachment to diverse shapes, sizes and substrates. Programmable actuation of hook deployment and geometry.
Aerial climbing roots from stem surface	For solidly attaching climbing stem to many kinds of support and surface. (step 2 attachment for secure attachment and anchoring, bracing for supporting further growth at apex).	Roots invading and adhering to surfaces and crevices (exact mechanisms under study).	Integrate artificial “secondary growth and attachment processes” e.g., “second step” attachment and adhesion following initial steadying. For ensuring support/bracing for growing robot body through unpredictable supports and voids.

### Life History and Terrain

Reaching across voids and efficient, secure attachment will be essential functions for growing robots that are designed to climb and thereby successfully negotiate three dimensionally unstructured, chaotic and even moving environments. Such a design strategy is interesting for applications where the targeted terrain is heterogeneous or not fully known. Potential applications that have been cited for such tasks include: reconnaissance and data gathering following landslides and earthquakes, archaeological investigations of buried/dangerous sites such as wells, ecological and agricultural measuring, surveillance in diverse and unpredictable habitats and space and planetary exploration and maintenance.

**Figure 7:**
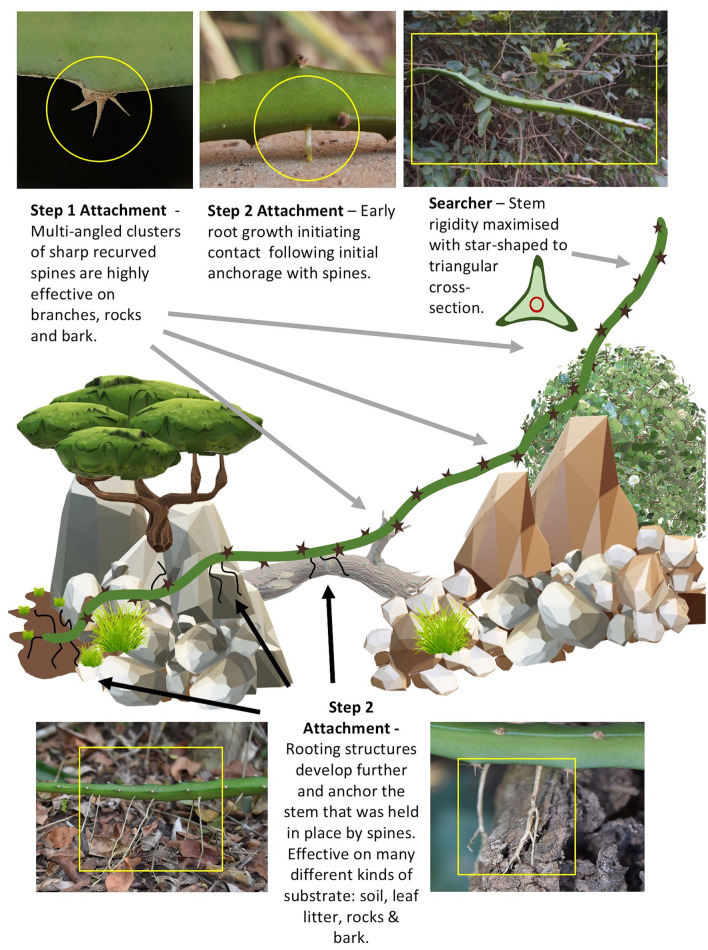
Depiction of growth mode and two-step attachment strategy of (*Selenicereus setaceus*) in traversing (roving, climbing and searching) through a highly unstructured environment with highly variable support substrates. Step 1: Multi-angled clusters of multi-angled recurved spines (top left) initiate preliminary anchoring (steadying) in relation to many different geometrical supports (flat, curved, large-thin cylinders, and substrates (hard rocks and cement, friable bark, leaves and soil). Stems held “in place” in otherwise potentially unstable positions during climbing and roving allow slower but more permanent attachment by roots to be initiated and make a first contact with a substrate (top center). Step 2: Roots grow and firmly anchor the stem to several kinds of substrates e.g., soil and leaf litter (lower left) and hard rocks/cement surfaces, tree surfaces such as bark (lower right). Grappling using spines is very effective especially in complex moving environments (like branches and leaves of other plants in the surrounding vegetation). Following initial and then firm attachment the stem can continue to explore across voids and uneven terrain but is now protected from falling away from its desired position as additive growth adds more mass and bending moments that would otherwise cause a fall. The searcher axes (top right) maximizes rigidity by developing a highly winged cross-section enabling the light-mass structure to cross voids of up to 1 m. It deploys further multi-angled hooks for further initial contact and anchoring to apparently most kinds of support in this habitat. These two mechanisms of attachment (fast and slow) ensure that this climbing plant (unlike many other climbing plants with higher support substrate specificity) can continuously grow and climb into a very wide range of habitats. The mechanisms therefore serve as a model for robotic artifacts that with design requirements for “highly diverse” support geometries and substrate properties.

Figure 7 summarizes some of the key features that contribute to this life-history at the habitat scale with a highly heterogeneous terrain. (A) Stems start growth rooted in the soil. (B) Early growth as circular to triangular stems can rove and climb across soil, rocks, and tree trunks using spines for initially attaching to these different substrates. (C) Searcher stems develop when the growing tip meets voids and stems develop an optimized geometry to increase rigidity. (D) A two-step attachment strategy ensures initial stabilization via hooks followed by strong attachment via fibrous adhesive roots.

We propose that useful insight for new innovations in robotics can be made by considering entire life-histories of different climbing plant species—how combinations of different functional traits integrate and are adapted for a specific kind of “robotic niche.” We think that this approach should be possibly just as useful as choosing specific functions and structures drawn from parts of a climbing plant life history. For example, the combination of robust (diameter in cm rather than mm), cheaply constructed searcher stems equipped with two kinds of attachment organ is evidently suited for extremely heterogeneous unstructured terrain, soil, rocks, tree trunks and fine branches and leaves with voids of up to 1.5 meters. Other kinds of climbing plant life history such as stem twining or tendril climbing will not be able to navigate and attach to such a heterogeneous range of supports since they are best adapted to attach to a 3-dimensional environment of cylinders (tree branches). It has long been known since Darwin's experiments and from ecological studies ever since that certain kinds of attachment are limited to certain kinds of support (Darwin, 1867; Peñalosa, 1982; Hegarty, 1991; Gianoli, 2015).

The climbing cactus shows a strong ability to grow through very unstructured and heterogeneous environments by attaching to a very wide range of supports and being able to cross voids of about a meter in length. We propose that these life history traits can provide possible functional innovations for designing robots that are required to navigate through unstructured terrains.

### Crossing Voids

Ensuring reach across spaces via growth represents a significant challenge for plants as well as robotic artifacts. Recent studies have made great progress in developing growing root-like robots for movement in soil (Sadeghi et al., 2014, 2016) and for movements comparable to climbing plant stems in air and in relation to supports (Del Dottore et al., 2019). These approaches require potentially different behaviors and technological innovations. Movements in and through soil media require negotiating and growth-by-bending around and between obstacles (rocks and solid substrates) and this has required adaptive behavior of the additive engineering process for artificial root lengthening (Sadeghi et al., 2014, 2017).

The searcher stems of the climbing cactus described here indicate that maximal rigidity using low density materials can be optimized by developing (a) a highly lobed cross-section rather than a more typical cylindrical organization in stems that need to cross-gaps (Figure 6) and a relatively “simple” combination of tissue layers with minimal secondary radial growth. This kind of organization provides a simple biological model for additive growth technologies (3D printing, electron spinning and expanding polymer, hydrogel or foam-based materials). It offers a way of modulating rigidity without complex secondary radial growth or the equivalent of longitudinally continuous fiber composite materials (the equivalents of wood and fiber tissue) for robotic “stems” to traverse gaps and voids to over a meter at this scale. If additive manufacturing technologies can construct different layers of materials having different mechanical properties such as a thin stiff outer skin and a bulking but light inner “tissue” in the forms of lobed cross-sections this would go some way to providing high rigidity for the minimum mass, as well as reducing problems of bending and torsion moments and “cost” of material production in terms of energy.

Our study did not enable us to resolve what might be the stimulus or trigger, either internal or extrinsic, that initiates geometrical and mechanical changes from circular to star-shaped. However, our observations of young “small” diameter individuals in well-lit forest floor situations suggest that light might be a factor governing this rather than ontogeny. It is also possible that the response might also be due to bending moments when growth exceeds the length of a support. Interestingly, lack of a support (presence of a void) and presence of light coincide in many natural situations here such as the edge of the forest or forest gaps. The shift to a winged cross-section optimizing light capture and rigidity as an adaptive response would be an interesting aspect to investigate more closely. It would also be of further interest to observe the behavior on encountering voids in dark situations.

Our observations indicated that low biomass stems are capable of navigating by “steering” toward, along, around and away from obstacles during first to second steps of the attachment phase (Figure 1E arrow 1, arrow 2). The ability to change direction while still retaining flexural stability is a crucial functional trait for searchers of climbing plants. In the cactus this likely depends on geometrical and material optimization below the growing apex. In artificial systems such changes will likely depend on the kind of growing mechanism—either immediately behind the apex such as apical additive manufacturing devices, or by other processes which influence (modify or add to) the stem after the “artificial” tissues have been formed. Ongoing studies on the *Selenicereus* anatomy, morphology and mechanics will investigate to what extent soft tissues modulate bending of the stem for adaptive growth (Box 1).

As pointed out above, one of the aims of developing movement by growth artifacts is the ability to negotiate navigate and climb through unstructured environments where the kinds of terrain are possibly far more heterogeneous than a system of cylindrical supports. Many climbing plants that do not fully twine can develop open hook-like stem curvatures that shape their surface for better adherence to broad surfaces like trees via micro-hooks or roots. The cactus described here appears to make such movements in relation to large diameter supports (Figure 1E). We suggest that this kind of “interactive growth” which does not require full twining might be an effective means by which “searching” robotic artifacts might align with encountered supports in order to deploy attachment mechanisms.

Finally, stems need to ensure rigidity and minimize mass in order to avoid dangerous bending moments that interfere with the desired directional growth via Euler buckling or worse sudden dramatic failure from local buckling. Artificial additive systems using an apical head are thus significantly end loaded and this is very different from most plant searchers in the biological world, which are nearly always tapered from base to apex with minimal end loading. This poses challenges for technologies using additive manufacturing if the mechanism (a) imparts significant end loading that increases bending and torsional moments or (b) is unable to mimic “secondary growth” of plants that adaptively increase rigidity below the apex. Keeping searcher stems light, rigid and optimized in terms of mass and end loading might mitigate against risks of buckling and enable search over wider voids.

### Two-Step Attachment

Unlike artificial root-like growth (Sadeghi et al., 2017) climbing, growing artifacts will possibly require alternative “adaptive” reactions in the transition from searching/spanning to attaching to “obstacles” rather than avoiding them (Walker, 2015). In the natural world there is probably not one single mode of attachment in climbing plants that will function in all habitats and on all supports. Attachment of growing robotic artifacts is a challenging prospect in environments with voids of different and unknown distances and with obstacles and supports of different, sizes, shapes, and surface properties. For example, stem twining and tendril twining organs are an appealing biological phenomenon for technical transfer e.g. (Must et al., 2019). However, among climbing plants, twining is highly constrained to the size and diameter of the support—i.e., slender, approximately cylindrical objects. Such mechanisms might not easily attach to flat or blocky objects or to friable or granular unconsolidated substrates. Climbing artifacts will likely need a more “generalized” or more “adaptive” system of attachment if they are to navigate through heterogeneous environments.

The cactus deploys a two-step attachment strategy (Figure 7) where clusters of sharp spines, are deployed on the stem in different orientations, each cluster bearing 3 to 5 spines, which are in turn deployed in different directions. This represents a multi-directional grappling system (Figure 7) and is effective on (i) narrow springy branches of shrubs and trees even in wind-blown environments, (ii) smooth or rough bark surfaces of trees, (iii) rock and concrete surfaces and (iv) soil and leaf litter surfaces. The engagement of sharp spines creates an initial grappling attachment that will prevent the stem from falling during continued growth and consequent shifts of weight distribution. It gives the plant “time” to deploy the slower growth of the root system to attach more firmly adjacent to the attached hooks (Figures 2 arrow 2, 7). The second step involves initialization of root growth from the stem followed by further root growth onto, into or around a large variety of supports differing in geometry and surface properties (soil, rock, on and around and within the crevices of bark surfaces).

The initial attachment is therefore “passive” (there is little or no active movement of spines following their deployment form the apex—but see below) with “pre-formed” structures (the spines are developed on the surface of the stem automatically and not in response to a nearby support or other stimulus such as gravity or shade). The second attachment by roots is an “active” growth process that needs to be triggered and its slow but secure attachment is relatively slow compared to the passive engagement of sharp spines. Passive and active attachment mechanisms are found widely throughout the biological diversity of climbing plants, they are also functionally linked to kinds of niche, especially the presence of short or wide voids and the arrangement and density of supports.

We suggest that multi attachment systems could be an invaluable design strategy for climbing artifacts, particularly in highly unstructured environments with extreme diversity of supports—just as we see in the natural range of situations for the climbing cactus (Box 1, Figure 7).

Hook and spine-inspired structures have been a rich and general source of bioinspiration for attachment strategies e.g., (Gorb, 2008; Voigt et al., 2012; Gallenmuller et al., 2015). Grappling devices for climbing plant-inspired robotic devices have already been implemented in continuum robotic artifacts that can grapple onto supports (Wooten and Walker, 2018) and help anchor the device and support it for its continued searching and functioning. The notion of bracing in robotics (Book et al., 1984; Walker, 2015) has probably been played out many times in the diversification of climbing plants at variable scales of organization. Mechanisms that promote “early adherence” and “steadying” of adjacent structures before more solid attachment are probably common in climbing plants and are probably of great importance especially in perturbed environments. Darwin commented on the probable “early” attachment of small radius twining stems that were efficient in windy conditions for a twining species (Darwin, 1867; Gianoli, 2015). Even much smaller -scale adhesion mechanisms probably entail multi-step mechanisms, for example in highly specialized sticky pads of *Parthenocissus tricuspidata* (Boston Ivy) where small hook structures probably steady the deployment of sticky pads near their support (Steinbrecher et al., 2011). New technologies may potentially play an important role in mimicking these multi-step mechanisms, which climbing plants use to ensure functionality in unstructured and perturbed environments (Box 1).

Spines of the cactus are forward pointing and in a “folded away” position near the searching apex (Figure 1D. Further below the apex they change in position to become recurved and multi-angled. Technologies now exist for fabricating and modulating artificial hook properties (stiffness) and geometries (angle of curvature) (Fiorello et al., 2018, 2019) using Direct Laser Lithography (DLL), micro molding of PDSM, among others, and incorporated nano particle actuation. Such possibilities might be feasible for “pre-formed” spine attachment on robotic bodies. Based on our observations of *Selenicereus* such technologies might be useful for deploying hook-like structures so as to avoid snagging on to obstacles during extensional growth and be deployed following extension and in the vicinity of supports (Wooten and Walker, 2018). This kind of hook movement prior to deployment for attachment is known in other climbing plants such as the highly effective acanthophylls (modified hook-like leaflets) in the climbing palms (Isnard and Rowe, 2008).

The root adhesion system in *Selenicereus* follows initial hook attachment and firmly anchors segments of stem to a range of support substrates. Root attaching mechanisms are well described for English ivy and also entail a multi-step mechanism of passive and active processes at the micro to nano scale (Melzer et al., 2010, 2012). To our knowledge, as yet there is no published account of “growth-like” technologies that could mimic this kind of attachment in either apical additive engineering growth artifacts or pre-formed, telescoping/everting and continuum technologies.

In *Selenicereus* the apex “decides” which stem geometry and which tissue properties are best adapted for the requirements at the apex. It also produces hooks in a “pre-deployed” geometry. As in many climbing plants “second step processes” including secondary radial growth of the wood cylinder and growth of attachment root meristems laterally are crucial for fine-tuning rigidity and deploying strong and stable growth-mediated attachment by roots. These second-step processes are absolutely crucial for maintaining reach, minimizing end-load effects and providing secure and safe rather than risky or temporary attachment when the plant has found a support. A challenge for growing robotic designs will be to integrate multi-step growth and attachment devices along the “grown” part of the artificial stem as in many climbing plants such as the climbing cactus described here. A fascinating breakthrough in climbing plant robotics would be to enable such “secondary growth” and “deployment functions,” either by pre-forming structures that could be actuated during later development (such as the spines here) or embed structure in the additive manufacturing process that can be later triggered and capable of extendable growth for attachment or modifying properties of the stem.

Further research on the spine architecture and their deployment as well as the strength and attachment properties of the *Selenicereus* root system will afford more information on how such traits can translated into technological concepts and technologies.

## Data Availability Statement

The datasets generated for this study are available on request to the corresponding author.

## Author Contributions

PS and NR both conceived of the project, carried out the field and technical research, conceived of technical modifications for mechanical measurements in the field, analyzed the data, and wrote the manuscript.

## Conflict of Interest

The authors declare that the research was conducted in the absence of any commercial or financial relationships that could be construed as a potential conflict of interest.
